# Strontium isotope (^87^Sr/^86^Sr) data from archaeological sites in Utah, USA

**DOI:** 10.1016/j.dib.2019.104571

**Published:** 2019-09-26

**Authors:** Spencer F.X. Lambert

**Affiliations:** Department of Anthropology, Southern Methodist University, Dallas, TX, USA

**Keywords:** Fremont, Utah, Strontium isotopes, Zooarchaeology

## Abstract

The information presented here includes the results of strontium isotope analysis on 75 baseline samples from nine Fremont sites in Utah. The baseline samples are of lagomorphs and rodents with limited foraging ranges. The baseline ranges for each site were calculated with two standard deviations. Also included are the raw strontium isotopic data for 30 large game samples from Wolf Village, a Fremont site in Utah. Additional data include a map showing the location of the sites in this study, box plots portraying the local ranges of nine Fremont sites in Utah, and an individual value plot comparing the Wolf Village large game samples to the strontium baseline for the site. These data compliment the discussions and interpretations found in “Identifying Strontium Baselines and Large Game Animal Trade at Fremont Sites through Strontium Isotope (^87^Sr/^86^Sr) Analysis” [1].

**Table:** Specifications Table

Subject	Archaeology
Specific subject area	Isotope Analysis
Type of data	Map Box Plots Individual Value Plot Tables CSV file
How data were acquired	Quadrupole Inductively Coupled Plasma Mass Spectrometer (Agilent 7500CE ICP-MS)
Data format	Raw Analyzed Comparison of Data
Parameters for data collection	Selected mandibles of the same side for each species to ensure there were no duplicate individuals tested for baselines. All samples were pretreated with 5% acetic acid and rinsed three times with quadruple de-ionized water
Description of data collection	Measured^87^Sr/^86^Sr ratios in teeth samples from Fremont sites to identify strontium baselines across Utah
Data source location	Wolf Village Site, Utah County, Utah; Woodard Mound Site, Utah County, Utah; Hinckley Mounds Site, Utah County, Utah; Nephi Mounds Site, Juab County, Utah; Five Finger Ridge Site, Sevier County, Utah; Icicle Bench Site, Sevier County, Utah; Nawthis Village Site, Sevier County, Utah; Parowan Site, Iron County, Utah; Paragonah Site, Iron County, Utah. Specimens were analyzed at the Strontium Isotope Geochemistry Laboratory at the University of Utah
Data accessibility	With the article
Related research article	Spencer F. X. Lambert Identifying Strontium Baselines and Large Game Animal Trade at Fremont Sites through Strontium Isotope (^87^Sr/^86^Sr) Analysis *Journal of Archaeological Science: Reports* 27: 101936

**Table:** 

**Value of the Data** • Data is useful to other scholars who are interested in strontium isotope studies in Utah • This data will allow data from future research to be compared to the already identified strontium baselines in Utah • The data shows potential complications with testing teeth from semi-aquatic mammals to identify regional baselines

## 1 Data

**Fig. 1:**
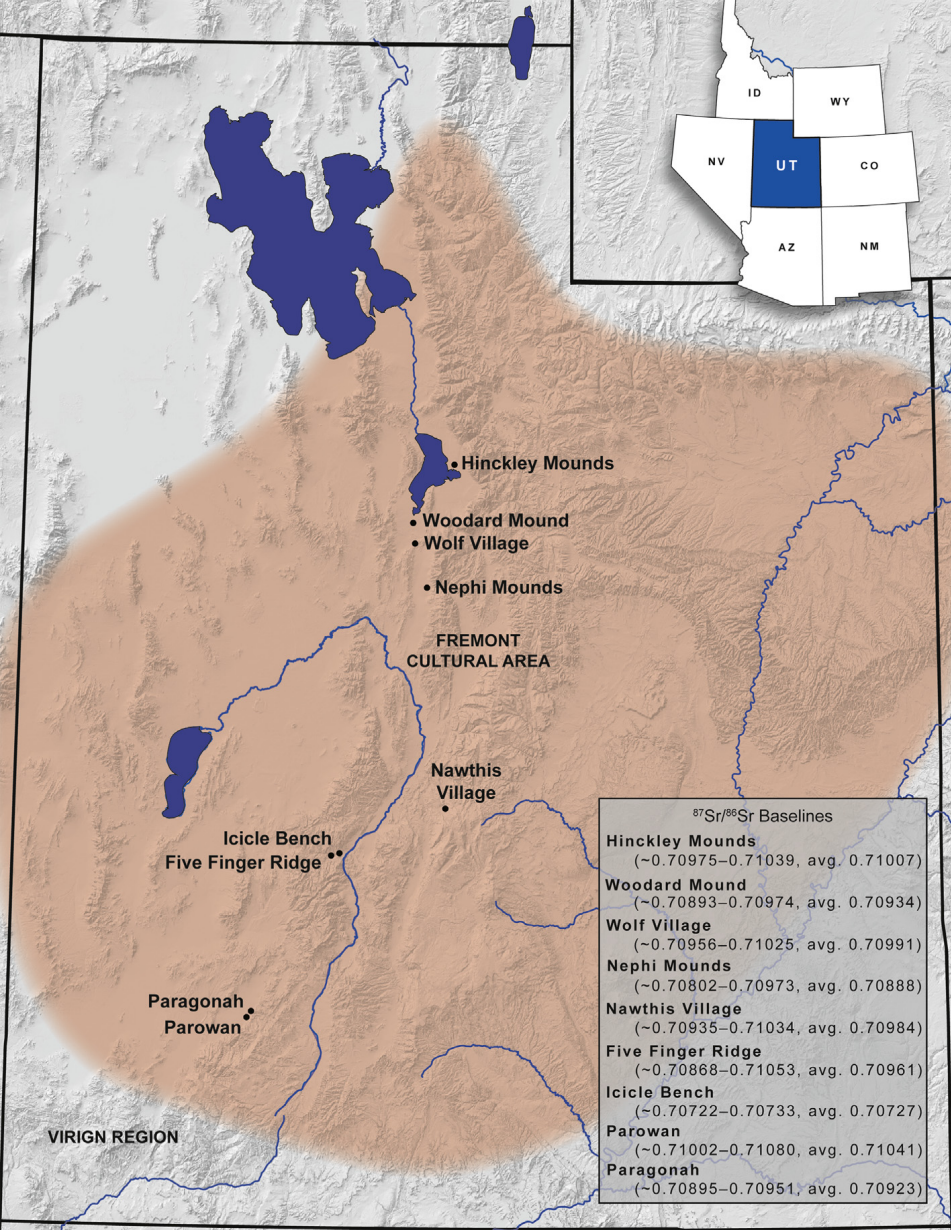
Map of Utah showing the location of the Fremont culture area and the sites included in this study. Map drafted by Scott Ure.

**Fig. 2:**
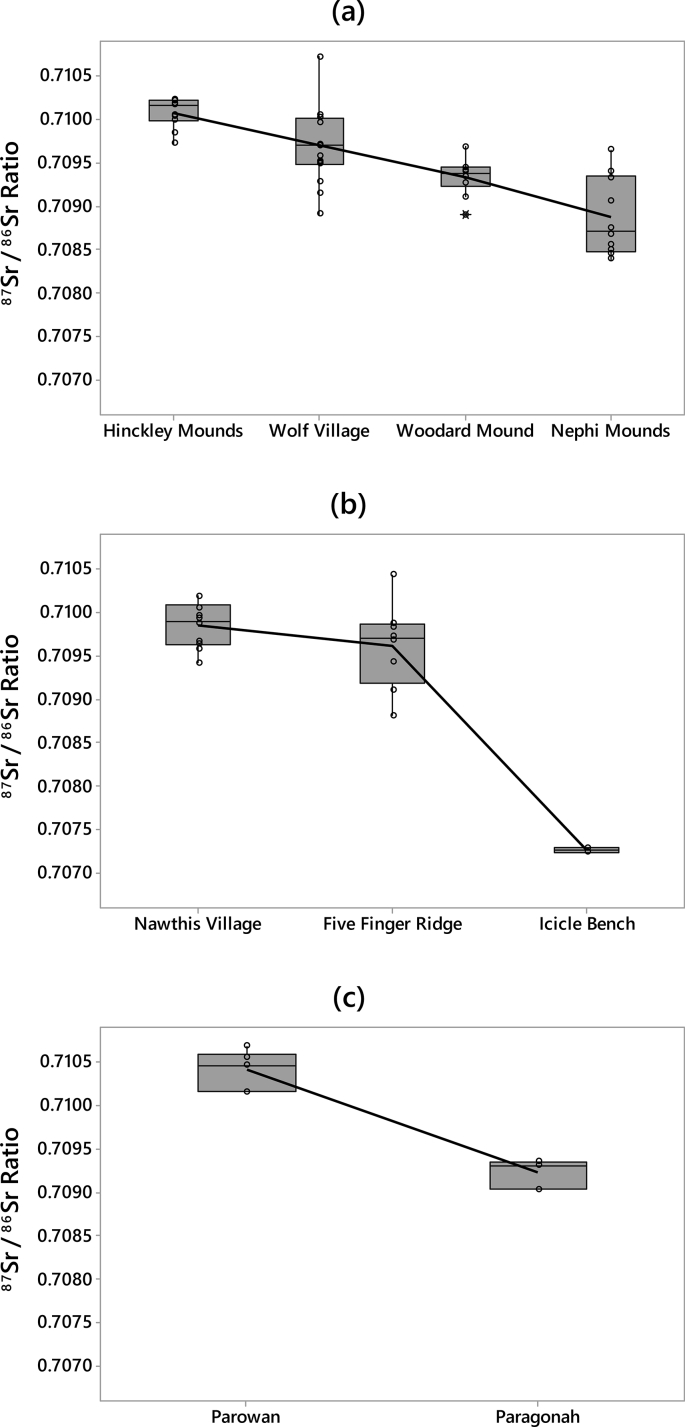
Boxplot of ^87^Sr/^86^Sr ratios for baseline samples from Fremont sites in: (a) northern Utah, (b) central Utah, and (c) the Parowan Valley.

**Fig. 3:**
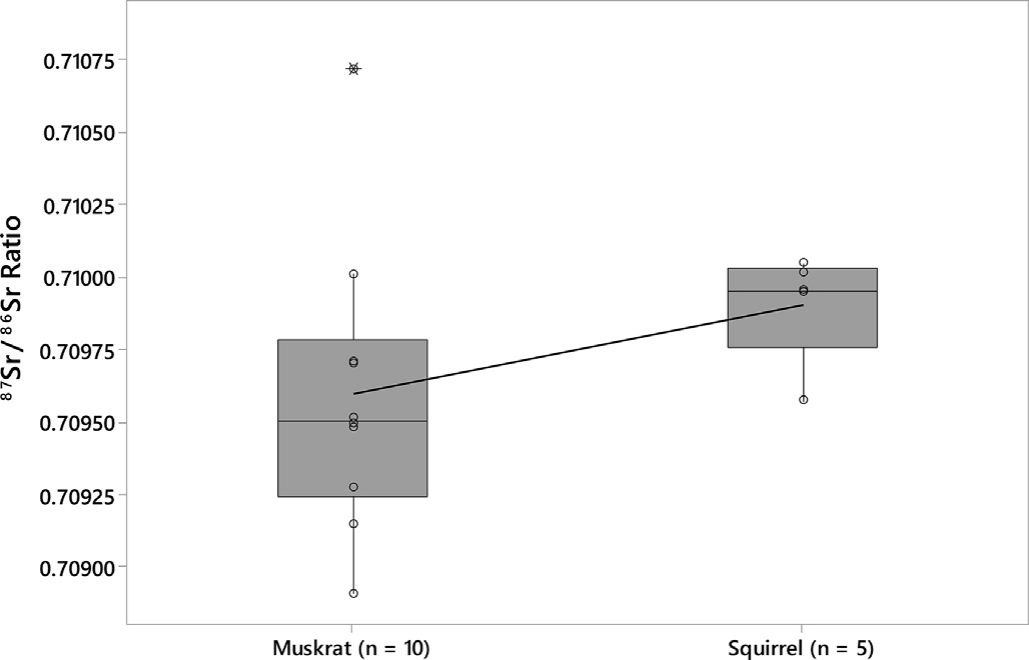
Boxplot of Wolf Village ^87^Sr/^86^Sr baseline ratios comparing the muskrat baseline to the squirrel baseline.

**Fig. 4:**
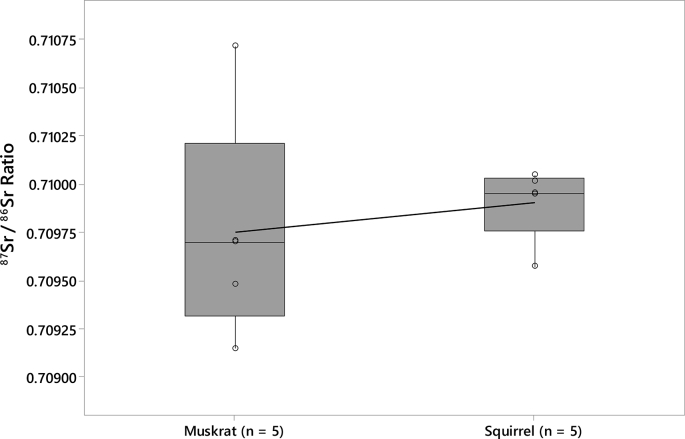
Boxplot of Wolf Village ^87^Sr/^86^Sr baseline ratios comparing a random sample of five muskrats to the squirrel baseline.

**Fig. 5:**
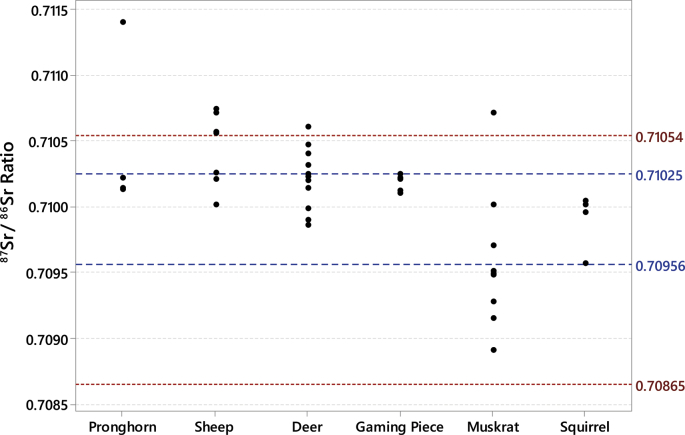
Individual value plot of Wolf Village ^87^Sr/^86^Sr ratios for large game, gaming pieces, muskrat, and squirrel compared to the Wolf Village baseline range based on squirrels (blue) and the baseline based on muskrats (red). Baseline range calculated with 2 standard deviations.

The data includes strontium isotope ratios (^87^Sr/^86^Sr) from 75 small mammal teeth from the archaeological record of nine Fremont sites in Utah (Table 1; see Fig. 1 for regional map and local baseline ranges for each of the sites). All small mammal teeth were analyzed to identify strontium baselines in their respective areas. Other data includes strontium isotope ratios (^87^Sr/^86^Sr) from 30 large game samples from the Wolf Village site (Table 2). The baseline and large game sample data are included in a supplemental CSV file. Box plots of local ranges for the nine Fremont sites indicates that local strontium ranges overlap at some areas far apart from one another and vary between some sites in close proximity (Fig. 2). The Wolf Village baseline was previously identified using muskrat incisors [1,2]. Recent strontium data using squirrel incisors from the same site indicate that small land mammals provide more precise baselines than semi-aquatic mammals such as muskrats (Fig. 3, Fig. 4, Fig. 5).

**Table 1:** Raw strontium (^87^Sr/^86^Sr) data for baseline samples from nine Fremont sites in Utah.

Sample ID	Site No.	Site Name	87Sr/86Sr	Error	Catalog No.	Taxa	Side
1494-31	42SV633	Nawthis Village	0.70967	0.000004	42SV633FS1292.3	*Sylvilagus* sp.	Right
1494-32	42SV633	Nawthis Village	0.70942	0.000004	42SV633FS3074.101	*Sylvilagus* sp.	Right
1494-33	42SV633	Nawthis Village	0.71018	0.000005	42SV633FS3208.1	*Sylvilagus* sp.	Right
1494-34	42SV633	Nawthis Village	0.71018	0.000005	42SV633FS3214.32	*Sylvilagus* sp.	Right
1494-35	42SV633	Nawthis Village	0.70957	0.000005	42SV633FS3112.11	*Sylvilagus* sp.	Right
1494-36	42SV633	Nawthis Village	0.70995	0.000004	42SV633FS2956.5	*Sylvilagus* sp.	Right
1494-37	42SV633	Nawthis Village	0.71004	0.000004	42SV633FS3028.1	*Sylvilagus* sp.	Right
1494-38	42SV633	Nawthis Village	0.70992	0.000005	42SV633FS2878.1	*Sylvilagus* sp.	Right
1494-39	42SV633	Nawthis Village	0.70987	0.000004	42SV633FS1680.1	*Sylvilagus* sp.	Right
1494-40	42SV633	Nawthis Village	0.70964	0.000005	42SV633FS2878.1	*Sylvilagus* sp.	Right
1494-41	42JB02	Nephi Mounds	0.70849	0.000003	42JB02FS12769	*Ondatra zibethicus*	Left
1494-42	42JB02	Nephi Mounds	0.70941	0.000004	42JB02FS823.13	*Lepus* sp.	Right
1494-43	42JB02	Nephi Mounds	0.70845	0.000004	42JB02FS12739	*Ondatra zibethicus*	Left
1494-44	42JB02	Nephi Mounds	0.70855	0.000005	42JB02FS825.1	*Sylvilagus* sp.	Right
1494-45	42JB02	Nephi Mounds	0.70965	0.000004	42JB02FS711.50	*Lepus* sp.	Right
1494-46	42JB02	Nephi Mounds	0.70875	0.000005	42JB02FS460.1	*Ondatra zibethicus*	Left
1494-47	42JB02	Nephi Mounds	0.70867	0.000004	42JB02FS12880	*Ondatra zibethicus*	Left
1494-48	42JB02	Nephi Mounds	0.70839	0.000003	42JB02FS258.25	*Ondatra zibethicus*	Left
1494-49	42JB02	Nephi Mounds	0.70932	0.000004	42JB02FS12859	*Lepus* sp.	Left
1494-50	42JB02	Nephi Mounds	0.70906	0.000004	42JB02FS680.77	*Ondatra zibethicus*	Left
1494-51	42IN100	Parowan	0.71055	0.000004	42IN100FS283.5	*Sylvilagus* sp.	Left
1494-52	42IN100	Parowan	0.71015	0.000004	42IN100FS509.1	*Sylvilagus* sp.	Left
1494-53	42IN100	Parowan	0.71067	0.000004	42IN100FS238.3	*Sylvilagus* sp.	Left
1494-54	42IN100	Parowan	0.71015	0.000004	42IN100FS283.4	*Sylvilagus* sp.	Left
1494-55	42IN100	Parowan	0.71046	0.000004	42IN100FS283.6	*Sylvilagus* sp.	Left
1494-56	42IN100	Parowan	0.71046	0.000005	42IN100FS433.9	*Sylvilagus* sp.	Left
1494-57	42UT273	Wolf Village	0.70949	0.000004	2016.010.16548.000	*Ondatra zibethicus*	Left
1494-58	42UT273	Wolf Village	0.70891	0.000004	2010.003.03565.015	*Ondatra zibethicus*	Left
1494-59	42UT273	Wolf Village	0.70928	0.000004	2012.002.10678.000	*Ondatra zibethicus*	Left
1494-60	42UT273	Wolf Village	0.70971	0.000004	2013.017.13518.000	*Ondatra zibethicus*	Left
1494-61	42UT273	Wolf Village	0.70951	0.000003	2013.017.13521.000	*Ondatra zibethicus*	Left
1494-62	42UT273	Wolf Village	0.70948	0.000003	2013.017.13678.000	*Ondatra zibethicus*	Left
1494-63	42UT273	Wolf Village	0.70970	0.000003	2016.010.16314.001	*Ondatra zibethicus*	Left
1494-64	42UT273	Wolf Village	0.71071	0.000003	2016.010.16692.030	*Ondatra zibethicus*	Left
1494-65	42UT273	Wolf Village	0.70915	0.000004	2012.002.09855.000	*Ondatra zibethicus*	Left
1494-66	42UT273	Wolf Village	0.71001	0.000004	2011.007.04942.004	*Ondatra zibethicus*	Left
1494-67	42IN43	Paragonah	0.70904	0.000004	42IN43125.8251	*Sylvilagus* sp.	Left
1494-68	42IN43	Paragonah	0.70931	0.000005	42IN43125.6798	*Sylvilagus* sp.	Left
1494-69	42IN43	Paragonah	0.70935	0.000004	42IN43125.8233	*Lepus californicus*	Left
1494-70	42UT102	Woodard Mound	0.70945	0.000004	1973.480.01175.614	*Ondatra zibethicus*	Left
1494-71	42UT102	Woodard Mound	0.70890	0.000005	1973.480.01179.252	*Ondatra zibethicus*	Left
1494-72	42UT102	Woodard Mound	0.70945	0.000005	1984.011.00457.000	*Ondatra zibethicus*	Left
1494-73	42UT102	Woodard Mound	0.70940	0.000004	1984.010.00246.004	*Ondatra zibethicus*	Left
1494-74	42UT102	Woodard Mound	0.70911	0.000004	1984.010.00246.010	*Ondatra zibethicus*	Left
1494-75	42UT102	Woodard Mound	0.70934	0.000004	1984.010.00246.000	*Ondatra zibethicus*	Left
1494-76	42UT102	Woodard Mound	0.70968	0.000004	1984.010.00246.000	*Ondatra zibethicus*	Left
1494-77	42UT102	Woodard Mound	0.70933	0.000004	1984.010.00246.000	*Ondatra zibethicus*	Left
1494-78	42UT102	Woodard Mound	0.70942	0.000003	1984.010.00246.000	*Ondatra zibethicus*	Left
1494-79	42UT102	Woodard Mound	0.70927	0.000003	1984.010.00246.000	*Ondatra zibethicus*	Left
1494-80	42UT111	Hinckley Mounds	0.71023	0.000003	2015.004.00218.004	*Ondatra zibethicus*	Left
1494-81	42UT111	Hinckley Mounds	0.71018	0.000003	2015.004.00218.001	*Ondatra zibethicus*	Left
1494-82	42UT111	Hinckley Mounds	0.71003	0.000003	2015.004.02463.010	*Ondatra zibethicus*	Left
1494-83	42UT111	Hinckley Mounds	0.70998	0.000002	2015.004.00086.004	*Ondatra zibethicus*	Left
1494-84	42UT111	Hinckley Mounds	0.70984	0.000003	2015.004.01983.002	*Ondatra zibethicus*	Left
1494-85	42UT111	Hinckley Mounds	0.71005	0.000003	2015.004.02441.001	*Ondatra zibethicus*	Left
1494-86	42UT111	Hinckley Mounds	0.71021	0.000003	2015.004.02310.012	*Ondatra zibethicus*	Left
1494-87	42UT111	Hinckley Mounds	0.71017	0.000004	2015.004.00192.007	*Ondatra zibethicus*	Left
1494-88	42UT111	Hinckley Mounds	0.71016	0.000002	2015.004.01075.001	*Ondatra zibethicus*	Left
1494-89	42UT111	Hinckley Mounds	0.70972	0.000004	2015.004.02397.005	*Ondatra zibethicus*	Left
1494-90	42UT111	Hinckley Mounds	0.71022	0.000003	2015.004.01007.004	*Ondatra zibethicus*	Left
1581-01	42UT273	Wolf Village	0.71005	0.000004	2011.007.06642.001	*Spermophilus* sp.	Left
1581-02	42UT273	Wolf Village	0.70957	0.000006	2012.002.08872.001	*Spermophilus* sp.	Left
1581-03	42UT273	Wolf Village	0.70995	0.000005	2012.002.09323.001	*Spermophilus* sp.	Left
1581-04	42UT273	Wolf Village	0.71002	0.000005	2012.002.09676.001	*Spermophilus* sp.	Left
1581-05	42UT273	Wolf Village	0.70995	0.000005	2012.002.09923.001	*Spermophilus* sp.	Left
1581-06	42SV1686	Five Finger Ridge	0.70983	0.000005	FIPR 13891	Lepus sp.	Left
1581-07	42SV1686	Five Finger Ridge	0.70967	0.000006	FIPR 13898	Lepus sp.	Left
1581-08	42SV1686	Five Finger Ridge	0.71042	0.000004	FIPR 13901	Neotoma sp.	Left
1581-09	42SV1686	Five Finger Ridge	0.70972	0.000005	FIPR 13938	Neotoma sp.	Left
1581-10	42SV1686	Five Finger Ridge	0.70910	0.000004	FIPR 13938	Neotoma sp.	Left
1581-11	42SV1686	Five Finger Ridge	0.70942	0.000005	FIPR 13938	Neotoma sp.	Left
1581-12	42SV1372	Icicle Bench	0.70724	0.000006	FIPR 14020	Neotoma sp.	Right
1581-13	42SV1372	Icicle Bench	0.70730	0.000004	FIPR 14037	Lepus sp.	Left
1581-14	42SV1686	Five Finger Ridge	0.70987	0.000004	FIPR 17076	Lepus sp.	Left
1581-15	42SV1686	Five Finger Ridge	0.70881	0.000005	FIPR 17111	*Mus musculus*	Right

**Table 2:** Raw strontium (^87^Sr/^86^Sr) data from Wolf Village (42UT273) large game samples.

Sample ID	87Sr/86Sr	Error	Catalog No.	Taxa	Element	Side	Weight (g)
1494-01	0.71022	0.000005	2016.010.16450.016	*Odocoileus hemionus*	3rd Molar	Right	0.07
1494-02	0.71014	0.000004	2012.002.09206.001	*Odocoileus hemionus*	3rd Molar	Right	0.07
1494-03	0.70990	0.000003	2013.017.13606.000	*Odocoileus hemionus*	2nd Molar	Right	0.06
1494-04	0.71047	0.000005	2012.002.10238.003	*Odocoileus hemionus*	3rd Molar	Right	0.08
1494-05	0.71020	0.000004	2013.017.13617.000	*Odocoileus hemionus*	1st Molar	Right	0.1
1494-06	0.71025	0.000004	2012.002.09148.001	*Odocoileus hemionus*	2nd Pre Molar	Right	0.05
1494-07	0.70999	0.000004	2010.003.03598.002	*Odocoileus hemionus*	2nd Pre Molar	Right	0.05
1494-08	0.70986	0.000006	2010.003.03448.003	*Odocoileus hemionus*	2nd Molar	Right	0.05
1494-09	0.71032	0.000004	2012.002.09206.002	*Odocoileus hemionus*	3rd Molar	Right	0.16
1494-10	0.71020	0.000004	2012.002.10230.002	*Odocoileus hemionus*	2nd Pre Molar	Right	0.1
1494-11	0.71040	0.000005	2011.007.07286.002	*Odocoileus hemionus*	1st Molar	Right	0.07
1494-12	0.71060	0.000005	2012.002.08835.001	*Odocoileus hemionus*	2nd Molar	Right	0.12
1494-13	0.71025	0.000004	2013.017.13536.000	*Odocoileus hemionus*	2nd Molar	Right	0.08
1494-14	0.71014	0.000004	2016.010.16664.004	*Antilocapra americana*	3rd Molar	Right	0.1
1494-15	0.71140	0.000005	2011.007.06929.001	*Antilocapra americana*	1st Molar	Right	0.11
1494-16	0.71022	0.000004	2016.010.15568.001	*Antilocapra americana*	3rd Molar	Left	0.16
1494-17	0.71013	0.000004	2011.007.07286.003	*Antilocapra americana*	1st Molar	Right	0.05
1494-18	0.71021	0.000004	2016.010.15568.002	*Ovis canadensis*	3rd Molar	Right	0.17
1494-19	0.71074	0.000004	2010.003.02733.001	*Ovis canadensis*	3rd Molar	Left	0.09
1494-20	0.71001	0.000005	2010.003.03496.007	*Ovis canadensis*	Molar	Right	0.08
1494-21	0.71071	0.000005	2012.002.10401.000	*Ovis canadensis*	Molar	Unknown	0.05
1494-22	0.71057	0.000004	2012.002.10238.002	*Ovis canadensis*	2nd Molar	Right	0.13
1494-23	0.71025	0.000004	2013.017.12292.000	*Ovis canadensis*	Molar	Unknown	0.1
1494-24	0.71021	0.000005	2013.017.13621.000	*Ovis canadensis*	Molar	Unknown	0.06
1494-25	0.71055	0.000005	2013.017.12602.000	*Ovis canadensis*	2nd Molar	Left	0.12
1494-26	0.71024	0.000005	2012.002.10276.002	Artiodactyla	Long Bone	N/A	0.16
1494-27	0.71012	0.000005	2012.002.11294.001	Artiodactyla	Long Bone	N/A	0.07
1494-28	0.71021	0.000005	2016.010.14673.002	Artiodactyla	Long Bone	N/A	0.07
1494-29	0.71021	0.000005	2011.007.07959.002	Artiodactyla	Long Bone	N/A	0.06
1494-30	0.71011	0.000005	2012.002.09177.001	Artiodactyla	Long Bone	N/A	0.09

## 2 Experimental design, materials, and methods

### 2.1 Sampling strategy

The purpose of the baseline samples was to identify strontium local ranges for three main areas in Utah, including: (1) the regions around Utah Lake, (2) central Utah, and (3) the Parowan Valley in southeastern Utah. All areas had large Fremont settlements. Sites around Utah Lake include Hinckley Mounds (42UT111), Woodard Mound (42UT102), and Wolf Village (42UT273). In addition, Nephi Mounds (42JB02) is somewhat close to the Utah Lake sites and could be accessed by the Fremont through Goshen Canyon. Sites in central Utah include Nawthis Village (42SV633), Five Finger Ridge (42SV1686), and Icicle Bench (42SV1372). Sites in the Parowan Valley in southeastern Utah include Parowan (42IN100) and Paragonah (42IN43). The multiple sites in each of the three main areas can be compared to identify variability in local strontium levels.

To avoid contamination from modern fertilizers and air pollutions, rodents and lagomorphs from the archaeological record are useful to identify local ^87^Sr/^86^Sr ratios [[3], [4], [5], [6]]. In this study, all baseline samples are from local small game. Lagomorph specimens were more common at dryland sites in central and southwestern Utah (i.e., Nawthis Village, Parowan, and Paragonah) and muskrat samples more common at wetland sites in northern Utah (i.e., Hinckley Mounds, Woodard Mound, Wolf Village, and Nephi Mounds) (Table 1). The initial goal for this project was to test ten samples from seven sites, but limitations on suitable specimens from Five Finger Ridge and the Parowan site required that additional specimens from nearby sites also be tested (i.e., Icicle Bench and Paragonah respectively). This was done with the assumption that sites near one another (<5 km apart) would share similar strontium baseline ranges. Thus, the local baselines for the Five Finger Ridge, Icicle Bench, Parowan, and Paragonah sites were all identified with less than ten samples.

### 2.2 Sample description

Seventy-five small game samples were analyzed to identify strontium baselines at nine sites. All baseline samples were incisors taken from intact mandibles from the archaeological record of each respective site in this study. To ensure all samples were from different individuals, all teeth from the same species were removed from mandibles of the same side. In addition, 30 large game samples from the Wolf Village site were also analyzed. These include 13 deer (*Odocoileus hemionus*) specimens, four pronghorn (*Antilocapra americana*) specimens, eight bighorn sheep (*Ovis canadensis*) specimens, and five worked bone gaming piece specimens constructed from long bones of unidentified large game (Artiodactyla) (Table 2). All tested large game specimens are from Fremont contexts. Like with the baseline samples, all deer samples were extracted from intact mandibles of the same side. Due to limited pronghorn and bighorn sheep specimens, a tooth sample from all suitable mandibles were analyzed so it is possible some are from the same individual.

### 2.3 Pre-treatment and strontium isotope analysis

All small mammal and large game samples were analyzed at the Strontium Isotope Geochemistry Laboratory at the University of Utah. Pretreatment was done at the Biogeochemistry Laboratory at the University of Utah. Small mammal samples are too small to manually remove dentine from enamel, so whole tooth specimens were analyzed. For large game samples, teeth were extracted from the mandible using a Dremel Lithium-Ion cordless drill (10.8 V, Model 800). Samples were examined under a Bausch & Lomb StereoZoom 5 (zoom range 0.8×–4.0×) microscope to ensure the dentine and discoloration were removed from the samples, leaving as much tooth enamel as possible (at least 0.05 g). The Dremel Lithium-Ion drill was also used to remove at least 0.05 g from each of the worked bone gaming pieces.

Samples were pretreated with 5% acetic acid (CH_3_COOH) and then rinsed three times in quadrupole de-ionized water (4 × H_2_O). These methods are effective at removing contaminants from samples [[7], [8], [9], [10]]. Next, samples were digested with cold nitric acid (HNO_3_) in sterile Teflon vials. Strontium concentrations were determined by analyzing the digested samples in a quadrupole inductively coupled plasma mass spectrometer (ICP-MS) (Agilent 7500ce, Santa Clara, CA). To isolate strontium from other ions, a small portion of the digest (200 ng) was purified using column chromatography with resin Sr-Spec (Eichrom, Lisle, IL) in an automated system (PrepFAST MC, Elemental Scientific, Omaha, NE). The purified Sr fraction was dried and then rehydrated with 1 mL of 5% HNO_3_. The samples were then analyzed on a Neptune Plus multi-collector ICP-MS (Thermo Scientific, Bremen, Germany). A certified reference material NIST (National Institute of Standard and Technology, Gaithersburg, MD) SRM 987 was run every three samples. A blank was run after each SRM sample with the SRM value (^87^Sr/^86^Sr = 0.71028) being within the acceptable range of other analysts [11].

### 2.4 Data analysis

Local ranges for nine Fremont sites were calculated with two standard deviations (Table 3). A box plot visual displays the homogeneity of strontium baselines at sites in northern Utah (Fig. 2a). While Nephi Mounds and Hinckley Mounds both vary, strontium values overlap between Wolf Village and the other three sites in northern Utah. Likewise, strontium values from sites in central Utah overlap with one another, specifically Nawthis Village and Fiver Finger Ridge. In contrast, sites close to one another such as Five Finger Ridge and Icicle Bench are highly variable (Fig. 2b). Similarly, both sites in the Parowan Valley have highly variable strontium baseline values despite their close proximity to one another (Fig. 2c).

**Table 3:** Strontium Baseline Ranges for Nine Fremont Sites measured with two standard deviations. Note the two Wolf Village baselines identified through either muskrats or squirrel samples.

Site No.	Site Name	No. Samples	Mean	SD	Local Range
42UT111	Hinckley Mounds	11	0.71007	0.00016	0.70975–0.71039
42UT102	Woodard Mound	10	0.70934	0.00020	0.70893–0.70974
42UT273	Wolf Village (muskrats)	10	0.70960	0.00047	0.70865–0.71054
42UT273	Wolf Village (squirrels)	5	0.70991	0.00017	0.70956–0.71025
42JB02	Nephi Mounds	10	0.70888	0.00043	0.70802–0.70973
42SV633	Nawthis Village	10	0.70984	0.00025	0.70935–0.71034
42SV1686	Five Finger Ridge	8	0.70961	0.00046	0.70868–0.71053
42SV1372	Icicle Bench	2	0.70727	0.00003	0.70722–0.70733
42IN100	Parowan	6	0.71041	0.00019	0.71002–0.71080
42IN43	Paragonah	3	0.70923	0.00014	0.70895–0.70951

Muskrat (*Ondatra zibethicus*) specimens may not reflect accurate strontium baselines since muskrats are semiaquatic animals. Muskrats may be influenced by nonlocal strontium coming from various geological formations through rivers and streams. Therefore, their bones and teeth may no longer represent local strontium baselines. Unfortunately, a previous baseline for Wolf Village was based on strontium levels in semiaquatic muskrat [2]. To test whether muskrat provide inaccurate or less precise strontium baselines than terrestrial mammals, five squirrel (*Spermophilus* sp.) incisors from Wolf Village were analyzed. The Wolf Village baseline using squirrels is more precise than the muskrat baseline and contains no outlier specimens outside the local strontium range (Fig. 3). Comparing a random sample of five muskrat specimens (Sample ID No. 1494-60, -62, -63, -64, -65) to the five squirrel specimens further indicates that the squirrel baseline is more precise (Fig. 4).

Comparisons between the muskrat and squirrel baselines to the Wolf Village large game specimens provide differing results (Fig. 5). Almost all large game samples fall within the muskrat baseline, indicating that most large game were local to the Wolf Village site. In contrast, the squirrel baseline suggests that approximately half the large game samples at Wolf Village were local to the area. In addition, most muskrat specimens fall outside the squirrel baseline suggesting that the muskrat samples are either of non-local individuals or no longer reflect local strontium signatures.

## Funding sources

Funding for this project was generously provided by the Charles Redd Center for Western Studies, the Grace Elizabeth Shallit Memorial Grant, the Warren Van Pelt Student Grant, and the Department of Anthropology at Brigham Young University. The Charles Redd Center for Western Studies funded the publication of this research.

## References

[1] Lambert S.F.X. (2019). Identifying strontium baselines and large game animal trade at Fremont sites through strontium isotope (^87^Sr/^86^Sr) analysis. J. Archaeol. Sci. Rep..

[2] Lambert S.F.X. (2018). Examining Large Game Utility and Transport Decisions by Fremont Hunters: a Study of Faunal Bone from Wolf Village, Utah. https://scholarsarchive.byu.edu/etd/6832/.

[3] Bentley R.A. (2006). Strontium isotopes from the Earth to the archaeological skeleton: a review. J. Archaeol. Method Theory.

[4] Bentley R.A., Price T.D., Stephan E. (2004). Determining the ‘local’ ^87^Sr/^86^Sr range for archaeological skeletons: a case study from Neolithic Europe. J. Archaeol. Sci..

[5] Malainey M.E. (2011). A Consumer's Guide to Archaeological Science: Analytical Techniques.

[6] Price T.D., Burton J.H., Bentley R.A. (2002). The characterization of biologically available strontium isotope ratios for the study of prehistoric migration. Archaeometry.

[7] Price T.D., Blitz J., Burton J.H., Ezzo J.A. (1992). Diagenesis in prehistoric bone: problems and solutions. J. Archaeol. Sci..

[8] Sillen A., Sealy J.C. (1995). Diagenesis of strontium in fossil bone: a reconsideration of Nelson et al. (1986). J. Archaeol. Sci..

[9] Thornton E.K. (2011). Reconstructing ancient Maya animal trade through strontium isotope (^87^Sr/^86^Sr) analysis. J. Archaeol. Sci..

[10] Valentine B., Kamenov G.D., Krigbaum J. (2008). Reconstructing Neolithic groups in Sarawak, Malaysia through lead and strontium isotope analysis. J. Archaeol. Sci..

[11] Copeland S.R., Sponheimer M., le Roux P.J., Grimes V., Lee-Thorp J.A., de Ruiter D.J., Richards M.P. (2008). Strontium isotope ratios (^87^Sr/^86^Sr) of tooth enamel: a comparison of solution and laser ablation multicollector inductively coupled plasma mass spectrometry. Rapid Commun. Mass Spectrom..

